# Should Benign Hepatic Tumours be Excised?

**DOI:** 10.1155/1991/84396

**Published:** 1991

**Authors:** A. K. C. Li, W. Y. Lau

**Affiliations:** Department of Surgery The Chinese University of Hong Kong Prince of Wales Hospital Shatin, NT Hong Kong


*HPB Surgery*, 1991, Vol. 4, pp. 251–253
Reprints available directly from the publisher
Photocopying permitted by license only

© 1991 Harwood Academic Publishers GmbH
Printed in the United Kingdom

HPB INTERNATIONAL

EDITORIAL & ABSTRACTING SERVICE

JOHN TERBLANCHE, EDITOR

Department of Surgery,

Medical School · Observatory 7925

Cape Town · South Africa

Telephone: (021) 47-1250 Ext. 229

Telefax (021) 47-8955

### SHOULD BENIGN HEPATIC TUMOURS BE EXCISED?

### ABSTRACT

*Iwatsuki, S., Todo, S. and Starzl, T.E. (1990) Excisional therapy for benign hepatic lesions. Surgery Gynecology & Obstetrics; 171: 240-246.*

**With the recent advances in imaging techniques, increased numbers of hepatic lesions are found today, and surgeons are asked frequently for the best course of management. Benign hepatic tumors sometimes cause life-threatening complications and more often trigger disabling or annoying symptoms in otherwise healthy individuals. Although various imaging techniques are quite accurate in identifying cysts and hemangiomas, other benign hepatic lesions, such as adenomas, focal nodular hyperplasia and other benign solid tumors, cannot be differentiated from malignant lesions with a high degree of confidence.**

### PAPER DISCUSSION

KEYWORDS: Benign hepatic tumour, liver resection

Iwatsuki and his colleagues reported the results of 219 hepatic resections for benign lesions out of 547 consecutive liver resections between October 1964 to June 1989. Only 5 patients died giving a 30 day hospital mortality of 2.3% and 14 patients (6.4%) developed post-operative complications.

Based on these excellent results, the authors advocated resections for benign lesions because of symptomatic relief, avoidance of haemorrhage from rupture and confirmation of the benign nature whenever diagnosis was uncertain.

In their experience, over 40% of all hepatic resections were for benign lesions. However achievement of technical excellence is not an argument for resection which remains the most effective therapy for primary hepatic malignancies. Since benign liver tumours have been found in up to 2% of patients in autopsy series[1], with widespread use of ultrasound investigations, detection of hepatic lesions will increase and surgeons will increasingly be consulted for management decisions.

If one can exclude conditions such as cysts, abscesses, tuberculomas, gummas and infiltrative or storage disorders which can be misdiagnosed as hepatic neoplasms[2] one can still be left with diagnostic uncertainty in a small minority of patients. This is particularly true in areas with high incidence of hepatocellular carcinoma (HCC) where this tumour is associated with post-hepatitis B cirrhosis. Despite radioactive isotope scanning, computerized tomography, magnetic resonance imaging and superselective angiography, the differentiation between a hyperplastic cirrhotic nodule and a small HCC can be difficult since both lesions give similar appearance on ultrasound[3] and selective angiography[4,5]. Hepatic intra-arterial injection of lipiodol combined with computerized tomography increases the sensitivity and specificity in the diagnosis of small HCC[3,6-8]. However false positive can still occur. Besides HCC, lipiodol uptake and retention have been reported in other primary[8] and secondary liver tumours[5,8], haemangiomas[4,5-8] focal nodular hyperplasia and nodular regenerative hyperplasia[8].

One differentiating characteristic is that the deposited lipiodol is cleared within a few weeks in a benign lesion while a malignant lesion will retain it for several months[5]. However to wait for weeks before a clinical decision can be made in patients with suspected malignancy, which may potentially be curable by liver resection, is unsatisfactory. We have found that lesions which retain lipiodol give a much brighter ultrasound image and facilitate ultrasound guided needle biopsy. To ensure that the biopsy obtained is in the area of interest, the sample must be stained for lipiodol, the technique of which we have now refined[9]. Despite the refinements in techniques of percutaneous needle biopsy under guidance of ultrasound, computerized tomography or laparoscopy, liver resections are still necessary in the management of dubious lesions.

Significant bleeding can occur in about 0.2% of percutaneous needle biopsies with a 0.1% mortality[10]. The risk of bleeding increases in patients with coagulopathy and in biopsy of vascular lesions like cavernous haemangioma as reported in this paper. Interestingly spontaneous rupture of adenoma which is a rare condition occurred in 6 out of 25 patients presenting as haemoperitoneum. In our locality 9.7% of HCC may rupture spontaneously and present as haemoperitoneum. Emergency resection, ligation of hepatic artery, plication of the bleeding points with packing have all been tried with limited success[11]. We now advocate superselective angiographic embolization if the patient's condition is reasonably stable or intratumoral injection of absolute alcohol to control haemorrhage[12] when confronted at laparotomy.

In the treatment of hepatic neoplasms the risks of liver resection have to be balanced against the quality and quantity of survival. In general, good risks patients with resectable malignant lesions should undergo liver resection as only surgery offers the hope of cure. For benign lesions the majority are small to moderate size and are usually asymptomatic. Once the diagnosis is confirmed, these should be observed and monitored with repeated ultrasound assessments, particularly in the first 2 years to avoid the potential danger of misdiagnosis. Liver cell adenoma, with a high incidence of spontaneous rupture and difficulty to differentiate from a well differentiated HCC on needle biopsy is a possible exception to this rule. For large symptomatic benign lesions or for those when malignancy cannot be excluded, the treatment of choice must still be hepatic resection. The rapid advances of liver surgery in the past two decades have made liver resection safe enough to advocate such a policy of treatment[13]. Experienced liver surgeons now report a mortality for major resection of less than 5% and even nil mortality is being reported in moderately sized series[14]. The good results reported by the authors of this paper further support this view.

It must be emphasized that the majority of benign lesions can be observed and that liver resection for benign conditions is not a minor undertaking. Good clinical results can only be obtained by surgeons experienced in liver surgery and by the team approach to the problem.

### REFERENCES

1. Henson, S.W.Jr., Gray, H.K., Dockerty, M.B. (1956) Benign tumours of the liver. *Surg. Gynecol. Obstet.*, **103**, 23–30
2. Schonland, M.M., Millward-Sadler, G.H., Wright, D.H., Wright, R. (1985) Hepatic Tumours. In: *Liver and Biliary Diseases*, (Ed.) Wright, Millward-Sadler, Alberti, Karran. 2nd ed. London: Balliere-Tindall. Ch. 41; pp. 1137–1184
3. Yumoto, Y., Jinno, K., Tokuyama, K., Araki, Y., Ischimitsu, T., Maeda, H., Konno, T., Iwamoto, S., Ohnishi, K., Okuda, K. (1985) Hepatocellular carcinoma detected by iodized oil. *Radiology*, **154**, 19–24
4. Okuda, K. (1986) Early recognition of hepatocellular carcinoma. *Hepatology*, **6**, 729–738
5. Sumida, M., Ohto, M., Ebara, M., Kimura, K., Okuda, K., Hirooka, N. (1986) Accuracy of angiography in the diagnosis of small hepatocellular carcinoma. *A.J.R.*, **147**, 531-536
6. Nakakuma, K., Tashiro, S., Hiraoka, T., Ogata, K., Ootsuka, K. (1985) Hepatocellular carcinoma and metastatic cancer detected by iodized oil. *Radiology*, **154**, 15-17.
7. Ohishi, H., Uchida, H., Yoshimura, H., Ohue, S., Ueda, J., Katsuragi, M., Matsuo, N., Hosogi, Y. (1985) Hepatocellular carcinoma detected by iodized oil. Use of anticancer agents. *Radiology*, **154**, 25–29
8. Bruneton, J.N., Kerboul, P., Grimaldi, C. (1988) Hepatic intraarterial lipiodol: Techniques, semiologic patterns, and value for hepatic tumours. *Gastrointest. Radiol.* **13** 45–51
9. Arnold, M.M., Wallace, A.C., Kreel, L., Li, K.W. (1990) Demonstration of lipiodol in paraffin sections using a modified silver impregnation technique. *Am. J. Clin. Pathol.* **94**, 585–9
10. Terry, R. (1952) Risks of needle biopsy of the liver. *Br. Med. J.*, 1102–1105
11. Dewar, G.A., Griffen, S.M., Ku, K.W., Lau, W.Y., Li, A.K.C. Management of bleeding liver tumours in Hong Kong. *Br. J. Surg.* (in press)
12. Chung, S.C.S., Lee, T.W., Kwok, S.P.Y., Li, A.K.C. (1990) Injection of alcohol to control bleeding from ruptured hepatomas. *Br. Med. J.* **301**, 421
13. Hardy, K.J. (1989) Liver Surgery: A revolution. *Aust. N.Z.J. Surg.* **59**, 519–521
14. Iwatsuki, S., Esquivel, C.O., Gordon, R.D., Starzl, T.E. (1986) Liver resection for metastatic cancer. *Surgery*, **100**, 804–810

A.K.C. Li
W.Y. Lau
Department of Surgery
The Chinese University of Hong Kong
Prince of Wales Hospital
Shatin, NT
Hong Kong

